# Unveiling pathogenesis of pelvic organ prolapse through transcriptomic and bioinformatic analyses in uterosacral ligament tissues of postmenopausal women

**DOI:** 10.3389/fgene.2025.1588278

**Published:** 2025-06-06

**Authors:** BingJie Rui, GuangHai Rui, YanFeng Yang

**Affiliations:** ^1^ Department of Obstetrics and Gynecology, Fushun Central Hospital, Fushun, Liaoning, China; ^2^ Department of Gynecology, CHA University, Seongnam, Gyeonggi, Republic of Korea; ^3^ Department of Obstetrics and Gynecology, Fushun Maternal and Child Health Hospital, Fushun, China

**Keywords:** pelvic organ prolapse, uterosacral ligament, non-coding RNA, transcriptome sequencing, FLJ20021

## Abstract

**Background:**

Pelvic organ prolapse (POP) is a common gynecological disorder arising from an imbalance in the synthesis and degradation of pelvic supportive tissues. Alterations in key molecules and genetic mutations affecting extracellular matrix (ECM) remodeling have been implicated in its development. This study aimed to profile coding and noncoding RNAs (ncRNAs) in uterosacral ligament tissues of postmenopausal women to elucidate POP’s molecular mechanisms.

**Methods:**

We enrolled five POP patients and three normal controls. Uterosacral ligament tissue samples were collected and analyzed using high-throughput transcriptome sequencing to profile messenger RNAs (mRNAs), micro RNAs (miRNAs), circular RNAs (circRNAs), and long noncoding RNAs (lncRNAs). Differential expression was determined using the criteria of |log_2_ (fold change)|>1 and an adjusted p-value (padj) < 0.05. Bioinformatics analyses, including Gene Ontology (GO) and Kyoto Encyclopedia of Genes and Genomes (KEGG) enrichment, were performed to assess the functional roles of the differentially expressed genes. Competing endogenous RNA (ceRNA) networks were constructed to explore interactions among lncRNAs, miRNAs, and mRNAs. Real-time quantitative polymerase chain reaction (qPCR) validated selected targets.

**Results:**

We identified 60 mRNAs, 146 miRNAs, 29 lncRNAs, and 176 circRNAs with significant differential expression in POP tissues. Functional enrichment analyses revealed that these transcripts are primarily involved in cellular senescence, inflammation, ECM regulation, and cytoskeletal organization. Several signaling pathways were enriched, including those mediated by mitogen-activated protein kinase (MAPK), Extracellular Signal-Regulated Kinase 1/2(Erk1/2), Ras-related proteins (Rap1), Forkhead Box O (FOXO), and other oncogene homologs. Analysis of ceRNA networks uncovered interactions among lncRNAs, miRNAs, and mRNAs. Notably, lncRNA FLJ20021 was significantly downregulated in POP tissues and correlated with altered expression of collagenⅢ (COL III), CollagenⅠ (COL I), and Matrix Metalloproteinase-9 (MMP9).

**Conclusion:**

Our findings demonstrate significant alterations in both coding and ncRNAs expression in POP tissues, suggesting that dysregulation of multiple pathways contributes to its pathogenesis. In particular, ECM remodeling and reduced FLJ20021 expression may play key roles in tissue degeneration, offering potential targets for future therapeutic intervention.

## Introduction

POP is primarily characterized by the descent of pelvic organs including the bladder, intestines, and uterus through the vaginal wall and pelvic floor, representing the predominant form of pelvic floor dysfunction (Smith et al., 2014). Epidemiological studies indicate that between 30% and 76% of women exhibit signs of POP during routine gynecological examinations (Barber, 2016), and its prevalence increases with age, with symptoms peaking around 61.5 years (Luber et al., 2001). Although it is widely acknowledged that up to 50% of women may experience prolapse, prevalence rates based on symptomatic criteria range from 3% to 6%, while vaginal examination findings can reach as high as 50%, with surgical intervention rates of approximately 1.5–1.8 per 1,000 women (Mattsson et al., 2020; Barber and Maher, 2013). Postoperative complications including persistent bladder and intestinal symptoms, sexual dysfunction, vault prolapse, and urinary incontinence remain problematic (Maher et al., 2013). The asymptomatic nature of early-stage POP, delayed treatment, and a high recurrence rate after surgery collectively contribute to significant social, physical, and mental burdens, ultimately diminishing quality of life, reducing labor productivity, and increasing healthcare costs (Slieker-ten Hove et al., 2009; Vergeldt et al., 2015; Wu et al., 2009).

Despite the increasing clinical prevalence of POP, its etiology and pathophysiology remain incompletely understood. In many diseases, ncRNAs have emerged as crucial regulators of gene expression, chromatin remodeling (Dai et al., 2025), and genomic stability (Hatzimanolis et al., 2025), with potential roles as diagnostic biomarkers, prognostic indicators, and therapeutic agents. In the context of POP, however, evidence linking lncRNAs to disease pathogenesis is still sparse. Recent studies have detected aberrant expression of various ncRNAs including miRNAs, lncRNAs, and circRNAs in POP‐affected tissues such as the uterosacral ligaments and vaginal walls (Zhang et al., 2024; Zhao et al., 2023). These alterations suggest that ncRNAs may influence key processes such as ECM remodeling, collagen metabolism, cellular apoptosis, and inflammatory responses, all of which are critical for maintaining the structural integrity of pelvic support (Jiang et al., 2024). Moreover, investigations into POP’s molecular mechanisms have identified dysregulation in several signaling pathways, including TGF-β, Wnt, and inflammatory cytokine cascades (Qin et al., 2020; Li et al., 2024). Consequently, elucidating the molecular mechanisms underlying the onset and progression of POP and identifying effective diagnostic targets are paramount for enhancing the prognosis and quality of life of patients with POP.

High-throughput sequencing technologies facilitate not only large-scale genome sequencing but also the analysis of gene expression, identification of non-coding RNAs, and selection of transcription factor target genes, thereby elucidating processes such as growth, differentiation, proliferation, apoptosis, and gene reprogramming, which are intimately associated with human diseases (Soon et al., 2013; Dillies et al., 2013). Recent findings indicate that approximately 3% of the human genome is transcribed into mRNA encoding proteins, while about 75% is transcribed into ncRNA, encompassing miRNA, lncRNA, and circRNA (Kimura, 2020). The differentially expressed ncRNAs are posited to play a pivotal role in diverse biological processes pertinent to the onset and progression of POP (Li et al., 2021), and can be identified through High-throughput Sequencing. In the present study, we employ high-throughput sequencing technology to perform a comprehensive transcriptomic analysis of uterosacral ligament tissues associated with POP. Our objectives are to systematically profile the expression of circRNAs, lncRNAs, miRNAs, and mRNAs and identify statistically and biologically significant differentially expressed transcripts using bioinformatics approaches. We aim to identify statistically and biologically significant differentially expressed transcripts, analyze the interactions and co-expression networks between coding and non-coding RNAs, and construct ceRNA networks. Furthermore, we will validate the differential expression of key lncRNAs at both tissue and cellular levels using real-time quantitative PCR, and assess their impact on extracellular matrix components—such as collagen and matrix metalloproteinases—in POP-related fibroblasts. This comprehensive approach is expected to yield novel insights into the molecular mechanisms underlying POP, thereby laying a foundation for improved early diagnosis, prognostic evaluation, and targeted therapeutic interventions.

## Materials and methods

### Patients and clinical specimens

Between January and December 2024, we conducted a prospective observational case–control study employing second-generation high-throughput whole transcriptome sequencing (RNA-Seq) of uterosacral ligament tissue at the Department of Gynecology, Shengjing Hospital, China Medical University (Shenyang, China).A total of 11 uterosacral ligament tissue specimens were collected from patients clinically diagnosed with pelvic organ prolapse (POP), based on the Pelvic Organ Prolapse Quantification System described by DeLancey (Bump et al., 1996). Additionally, six normal uterosacral ligament tissue specimens were obtained from patients without POP who underwent hysterectomy for benign cervical disease at the same institution. All patients were postmenopausal. We randomly selected 5 cases from the POP group and 3 cases from the control group for RNA-Seq, while the remaining samples were used for qPCR validation studies. This study was approved by the Institutional Ethics Committee of Shengjing Hospital, China Medical University (approval number: 2024PS020K) in accordance with the Declaration of Helsinki ethical guidelines. Basic demographic and clinical data for both groups, including age, body mass index (BMI), parity, duration of postmenopause, smoking habits, and medical history (including hormone replacement therapy, chronic cervicitis or vaginitis, malignancy, endocrine, and immune disorders), were recorded (Table 1).

**TABLE 1:** Comparison of demographic and clinical characteristics between the POP and control groups.

Variable	POP group (n = 11)	Control group (n = 6)	p-value
Parity, median	1	1	0.285
Age, mean ± SD, years	60.00 ± 4.796	57.67 ± 3.215	0.392857
BMI, mean ± SD, kg/m^2^	26.35 ± 3.403	27.50 ± 4.574	1.000000
Postmenopause, mean ± SD, years	9.6000 ± 3.91152	7.3333 ± 1.15470	0.250000
Hormone Replacement Therapy, n (%)	0	0	1.000000
Chronic Cervicitis/Vaginitis, n (%)	0	0	1.000000
History of Malignancy, n (%)	0	0	1.000000
Smoking Habit, n (%)	0	0	1.000000
Endocrine Diseases, n (%)	0	0	1.000000
Immune Disorders, n (%)	0	0	1.000000

Note: a) Descriptive data are presented as numbers (%), mean ± standard deviation, or median, as appropriate. b) Continuous variables (age, BMI, and duration of postmenopause) were compared using the Mann-Whitney U test, while categorical variables were analyzed using Fisher’s exact test. c) Postmenopause is defined as at least 1 year after cessation of menstruation. d) The history of endocrine diseases includes hypertension, thyroid disease, and diabetes. e) The history of immune disorders includes asthma, systemic lupus erythematosus, rheumatism, or osteoarthritis.

### RNA extraction and quality assessment

Total RNA was extracted from uterosacral ligament tissues using TRIzol™ (Invitrogen, Carlsbad, CA, United States). RNA integrity was evaluated using an Agilent 2,100 Bioanalyzer (Agilent Technologies, Santa Clara, CA, United States), ensuring RIN values ≥ 7. Concentration and purity were determined with a Qubit^®^ 3.0 Fluorometer (Life Technologies, CA, United States) and NanoDrop One spectrophotometer (Thermo Fisher Scientific, United States), respectively, with A260/A280 ratios around 2.0.

### Library construction and sequencing

Ribosomal RNA was depleted using the Ribo-Zero™ rRNA Removal Kit (Illumina, San Diego, CA, United States). Strand-specific cDNA libraries were constructed with the NEBNext^®^ Ultra™ Directional RNA Library Prep Kit for Illumina (NEB, United States). Libraries were sequenced in paired-end 150 bp mode on the Illumina NovaSeq 6,000 platform. Sequencing quality was assessed using FastQC, and low-quality reads were removed using Trimmomatic.

### Data alignment and expression quantification

High-quality reads were aligned to the human reference genome (GRCh38) using HISAT2. Gene expression was quantified with featureCounts, and normalized as FPKM. Differential expression analysis was performed using DESeq2 with padj < 0.05 and | log_2_ Fold Change| > 1.

### Functional enrichment analysis

Differentially expressed genes were subjected to GO, KEGG, and Reactome pathway enrichment analyses using the clusterProfiler package in R. The Benjamini–Hochberg method was used to padj.

### ceRNA network construction

Potential interactions among lncRNAs, circRNAs, miRNAs, and mRNAs were predicted using TargetScan, miRanda, and StarBase. The resulting ceRNA network was constructed and visualized with Cytoscape, highlighting key regulatory axes and the potential roles of non-coding RNAs in gene expression regulation.

### RNA extraction and qPCR

Total RNA was extracted from tissues and cells using TRIzol Reagent (Invitrogen) according to the manufacturer’s instructions. The extracted RNA was then reverse transcribed into complementary DNA (cDNA) using the PrimeScript^®^ RT Reagent Kit (Takara Bio, Japan) following the recommended protocol. qPCR was performed on an Applied Biosystems^®^ Real-Time PCR System (QuantStudio 3, Thermo Fisher Scientific, United States) using TB Green^®^ Premix Ex Taq™ II (Tli RNase H Plus) (Takara Bio, Japan) according to the manufacturer’s instructions. The thermal cycling conditions were as follows: an initial incubation at 95°C for 3 min, followed by 39 cycles of denaturation at 95°C for 10 s and annealing/extension at 60°C for 10 s. Relative gene expression was quantified using the 2^−ΔΔCT^ method. Primer sequences are listed in Table 2.

**TABLE 2:** The sequence of primer used in this study.

Primers	Sequence (5′–3′)
FLJ20021-fp	AAA​AGC​GGG​TCT​CCG​TCT​AC
FLJ20021-rp	AAC​CAC​GTT​GCC​AGT​CCT​TG
β-Actin-fp	GGG​AAA​TCG​TGC​GTG​ACA​TTA​AG
β-Actin-rp	TGT​GTT​GGC​GTA​CAG​GTC​TTT​G
COLⅠ-fp	GTG​CGA​TGA​CGT​GAT​CTG​TGA
COLⅠ-rp	CGG​TGG​TTT​CTT​GGT​CGG​T
COL Ⅲ-fp	GGA​GCT​GGC​TAC​TTC​TCG​C
COL Ⅲ-rp	GGG​AAC​ATC​CTC​CTT​CAA​CAG
MMP2-fp	CCC​ACT​GCG​GTT​TTC​TCG​AAT
MMP2-rp	CAA​AGG​GGT​ATC​CAT​CGC​CAT
MMP9-fp	TGT​ACC​GCT​ATG​GTT​ACA​CTC​G
MMP9-rp	GGC​AGG​GAC​AGT​TGC​TTC​T
TIMP1-fp	CTT​CTG​CAA​TTC​CGA​CCT​CGT
TIMP1-rp	ACG​CTG​GTA​TAA​GGT​GGT​CTG
TIMP2-fp	AAG​CGG​TCA​GTG​AGA​AGG​AAG
TIMP2-rp	GGG​GCC​GTG​TAG​ATA​AAC​TCT​AT

### Cell culture

Fresh uterosacral ligament tissues from the clinical samples were dissected into 1 × 1 × 1 mm pieces, distributed, and subsequently cultured in DMEM high glucose medium supplemented with 10% fetal bovine serum, 0.5 mg/mL streptomycin, 0.5 kU/mL penicillin, and 1.25 μg/mL amphotericin B, under a humidified atmosphere containing 5% CO_2_ at 37°C. Cells from the three to sixth generations were selected for experimentation.

### Immunofluorescence (IF) staining

Uterosacral ligament fibroblasts (passage 2–3) cultured on coverslips were fixed with 4% paraformaldehyde, permeabilized with 0.1% Triton X-100 for 20 min, and blocked with 3% BSA (or 10% donkey serum for goat-derived antibodies) for 30 min at room temperature. The cells were then incubated overnight at 4°C with primary antibodies. After three 5-min washes with PBS, a corresponding fluorophore-conjugated secondary antibody was applied for 50 min at room temperature in the dark. Following additional PBS washes, nuclei were counterstained with DAPI for 10 min, and coverslips were mounted using an anti-fade reagent. Fluorescent images were acquired using a Nikon Eclipse C1 microscope with appropriate filter settings. Primary antibodies used, supplied by Wuhan Servicebio Technology Co., Ltd., included: Vimentin (1:200, GB11192), Desmin (1:500, GB12075), and Pancytokeratin (1:500, GB122053).

### siRNA and plasmid transfection methods

Small interfering RNAs (siRNAs) targeting FLJ20021 was synthesized by RiboBio Co., Ltd. (Guangzhou, China) using sequence-specific complementary base pairing for gene silencing. The si-FLJ20021 (5′-GCA​ATA​GTG​GCC​CAA​ATG​T-3′) and si-NC(5′-UUCUCCGAACGUGUCACGUTT-3′). The cDNAs for FLJ20021were cloned into plasmid vectors for overexpression, with technical support provided by Hanbio Co., Ltd. (Shanghai, China). Transfection of siRNA and overexpression plasmids were performed according to the manufacturer’s instructions using Lipofectamine 3,000 Transfection Reagent (Thermo Fisher Scientific).

### Statistical analysis

Statistical analysis (presented as mean ± standard deviation) was performed using SPSS version 26.0 (IBM SPSS Inc., Chicago, IL, United States). Comparisons across two samples were conducted applying Student’s t-test, and a p-value<0.05 was seen as remarkable with respect to statistics. Graphs were created with GraphPad Prism 9.5.0.

## Results

Baseline demographic and clinical characteristics were comparable between POP patients and controls (Table 1). No significant differences were observed in parity, age, BMI, duration of postmenopause, or in the prevalence of hormone replacement therapy, chronic cervicitis/vaginitis, malignancy history, smoking habit, endocrine diseases, or immune disorders (all p > 0.05), confirming that the two groups were well matched.

### Differential expression analysis of lncRNAs, mRNAs, circRNAs, and miRNAs

**FIGURE 1:**
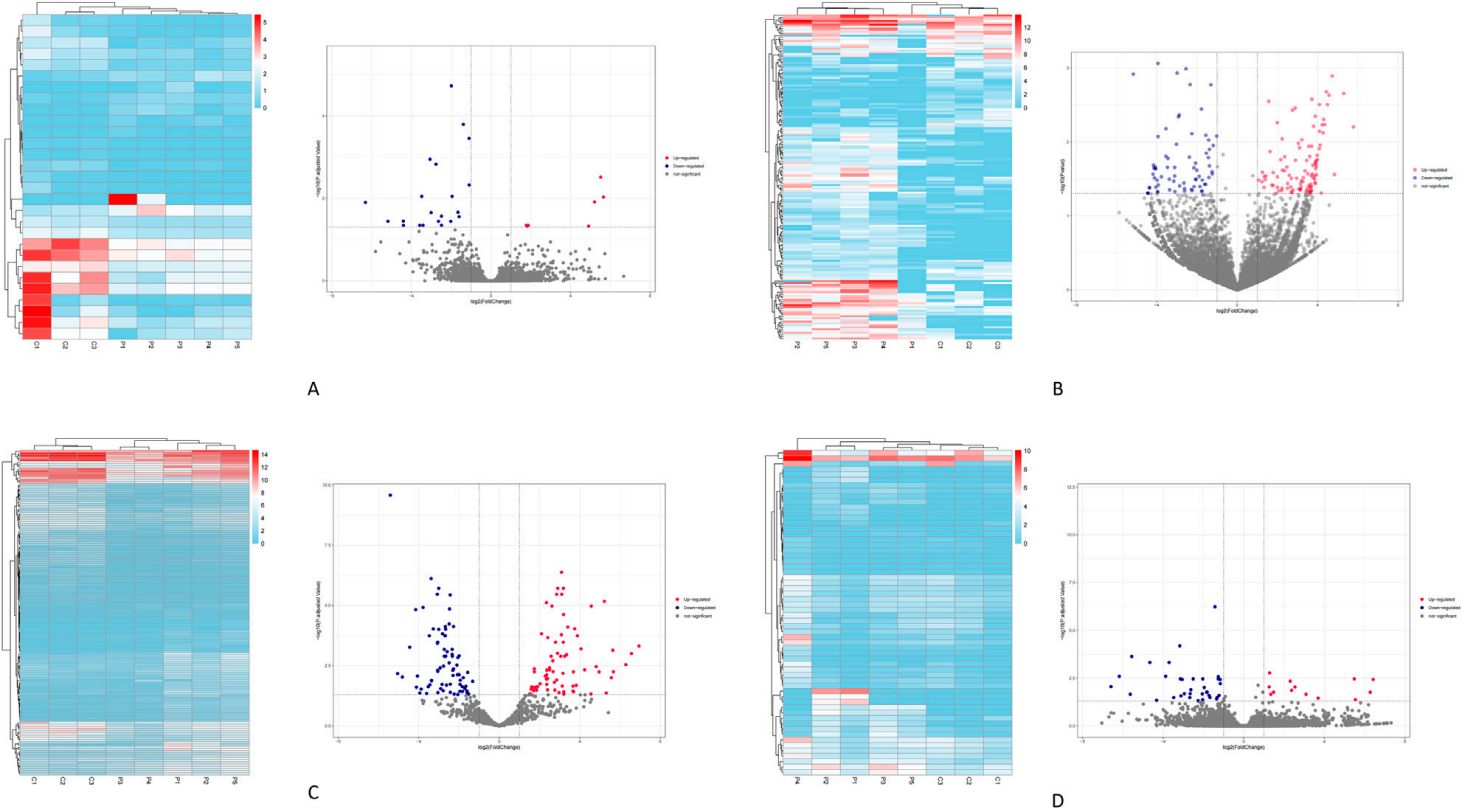
Illustrates the clustering heatmaps and volcano plots for the differentially expressed transcripts. **(A)** the lncRNAs. **(B)** the circRNAs. **(C)** the miRNAs. **(D)** the mRNAs. Red indicates transcripts that are upregulated, while blue indicates those that are downregulated.

Based on the screening criteria of |log_2_ (fold change) | > 1 and P-value <0.05, a total of 411 differentially expressed transcripts were identified. This includes 29 differentially expressed lncRNAs (8 upregulated and 21downregulated) (Figure 1A), 176 differentially expressed circRNAs (105 upregulated and 71 downregulated) (Figure 1B), 146 differentially expressed miRNAs (72 upregulated and 74 downregulated) (Figure 1C), and 60 differentially expressed mRNAs (25 upregulated and 35 downregulated) (Figure 1D).

### GO and KEGG enrichment analysis reveal biological processes and signaling pathways of POP

**FIGURE 2:**
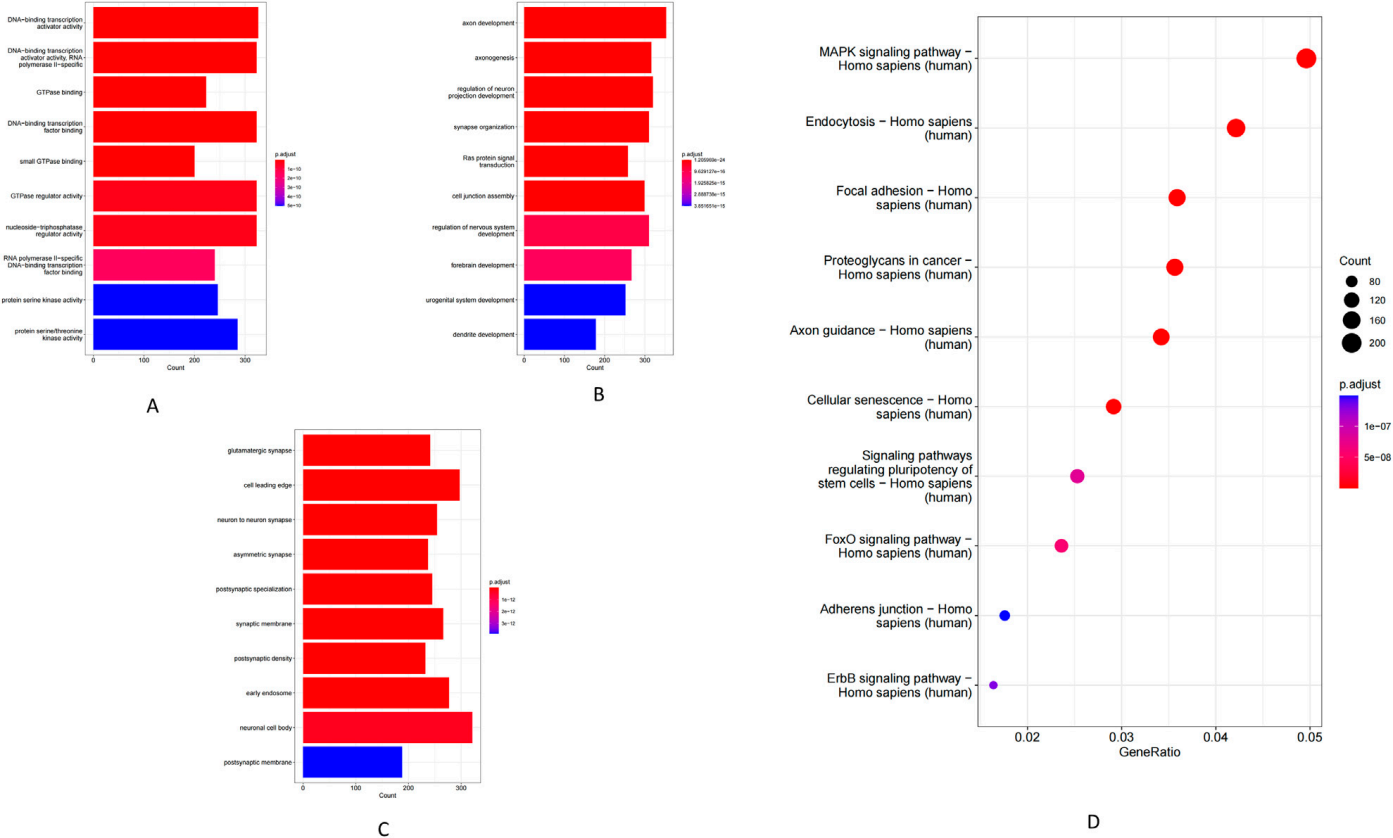
GO enrichment and KEGG pathway analysis of miRNAs. **(A)** Molecular function. **(B)** Biological process. **(C)** Cellular component. **(D)** KEGG pathway.

GO enrichment analysis of genes targeted by the differentially expressed miRNAs revealed significant enrichment in DNA-binding and GTPase activity under the Molecular Function (MF) (Figure 2A). Enriched Biological Processes (BP) terms included Ras protein signal transduction, axon guidance and synaptic function, and neuronal and urogenital system regulation (Figure 2B). Similarly, the prominent Cellular Component (CC) terms were largely neuronal structures–such as neuronal cell bodies, neuron-to-neuron (glutamatergic) synapses, and postsynaptic densities and membranes (Figure 2C). Consistent with these GO findings, KEGG pathway analysis showed involvement of the miRNA target genes in several related pathways. Notably, the top enriched KEGG pathways included axon guidance, focal adhesion, cellular senescence, autophagy, and proteoglycans in cancer, alongside key signaling cascades such as the FOXO and MAPK pathways (Figure 2D).

**FIGURE 3:**
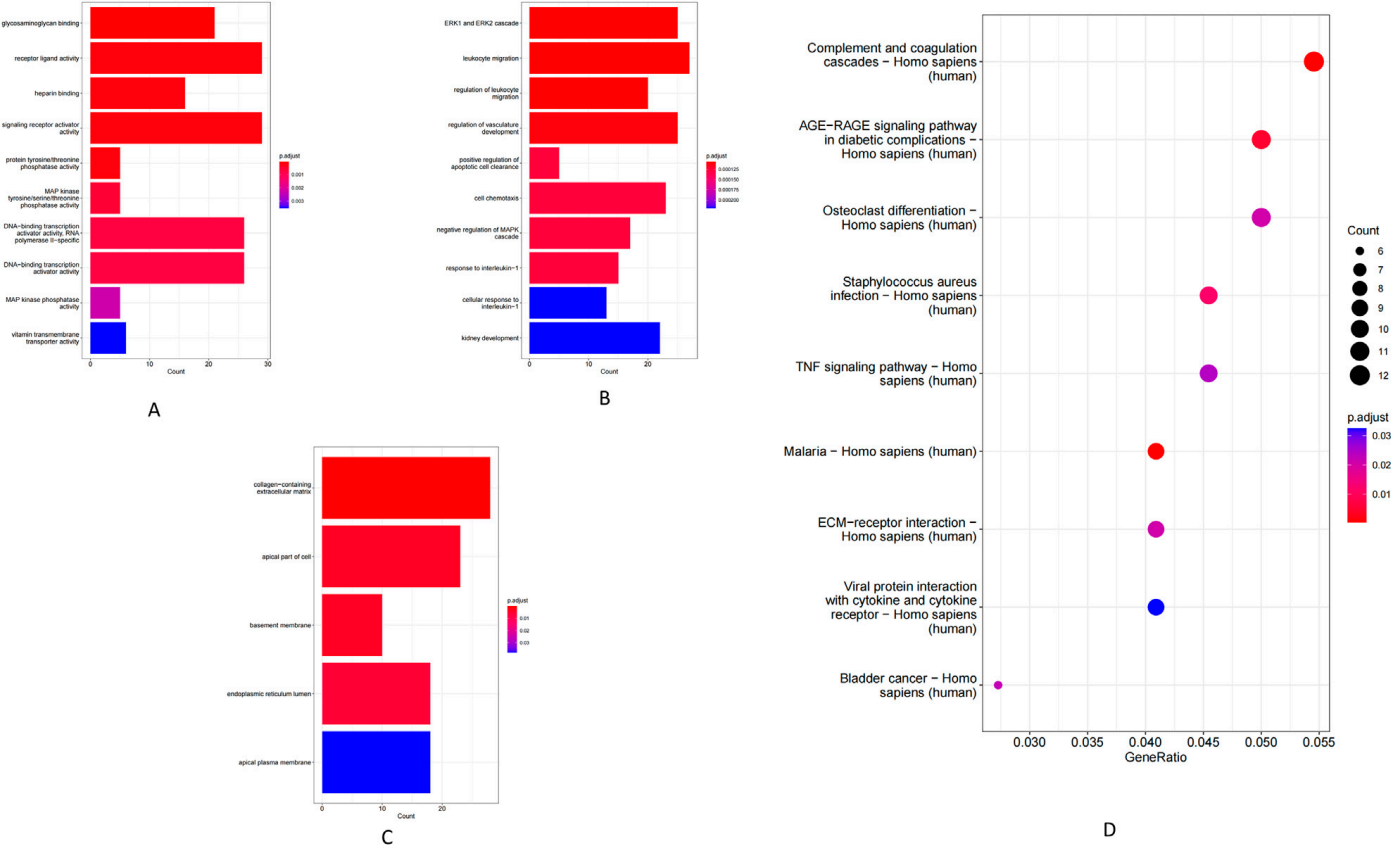
GO enrichment and KEGG pathway analysis of lncRNAs. **(A)** Molecular function. **(B)** Biological process. **(C)** Cellular component. **(D)** KEGG pathway.

Using co-localization (within 100 kb of each lncRNA) and co-expression (Pearson r > 0.98) criteria, we identified a set of lncRNA target genes for enrichment analysis. GO analysis showed that these target genes were significantly enriched in MF such as receptor–ligand activity, RNA polymerase II-specific transcription factor activity, and DNA-binding transcription activator activity (Figure 3A). The most enriched BP included leukocyte migration and MAPK/ERK cascade signaling (Figure 3B), while enriched CC were mainly extracellular matrix components (e.g., collagens) and the endoplasmic reticulum lumen (Figure 3C). Consistently, KEGG pathway analysis revealed significant enrichment of key immune and inflammatory pathways, including complement and coagulation cascades, TNF signaling, the AGE–RAGE signaling pathway, and extracellular matrix–receptor interactions (Figure 3D).

**FIGURE 4:**
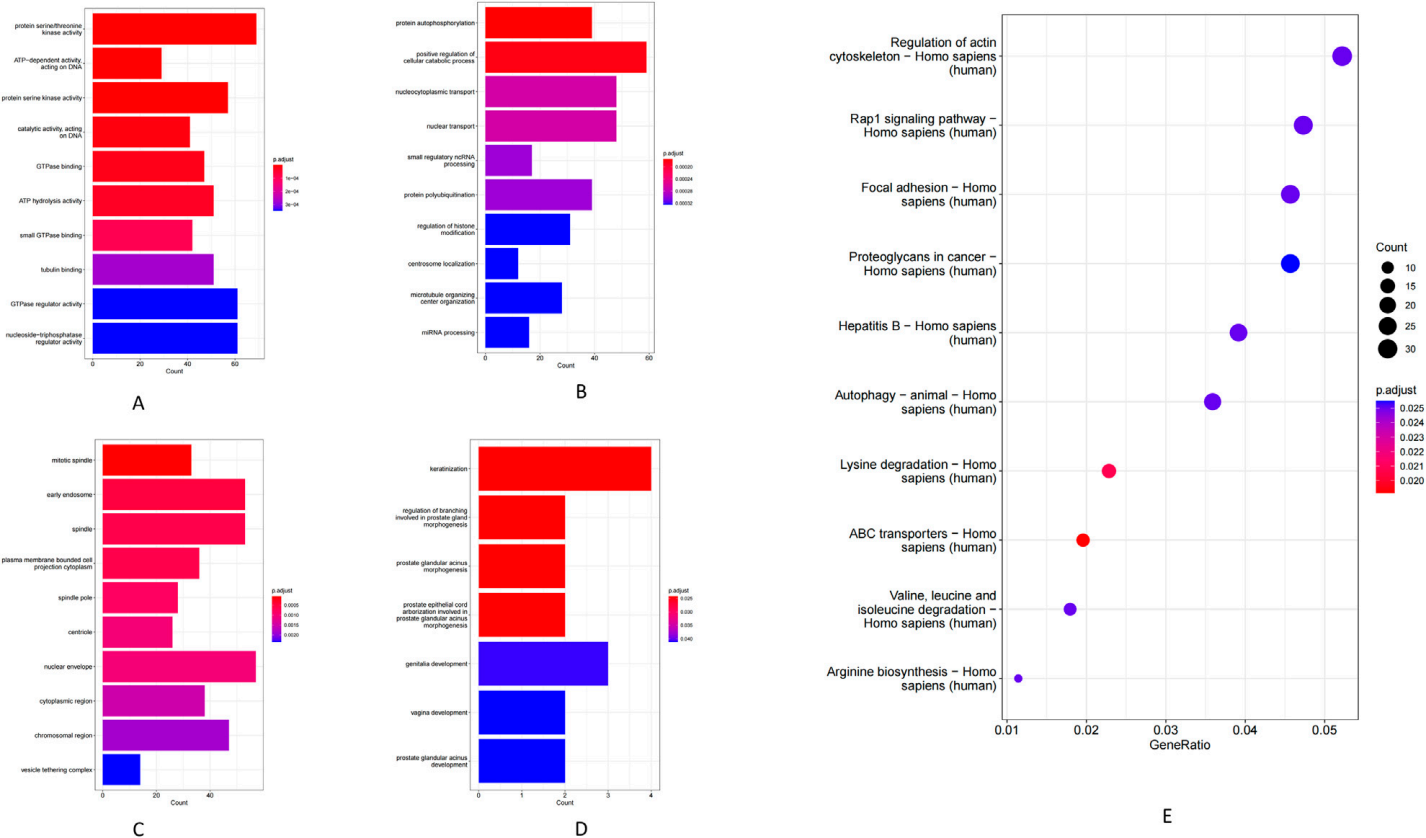
**(A)** Molecular function of circRNA-regulated genes. **(B)** Biological process of circRNA-regulated genes. **(C)** Cellular component of circRNA-regulated genes. **(D)** mRNA genes GO-BP. **(E)** KEGG pathway.

At the MF level, circRNA host genes were enriched for ATP hydrolysis activity, serine/threonine protein kinase activity, and GTPase binding (Figure 4A). Enriched BP included cellular catabolic metabolism, protein phosphorylation, and nucleocytoplasmic transport (Figure 4B). In the CC, these genes were mainly associated with the nuclear membrane, early endosomes, and the cytoplasm (Figure 4C). KEGG pathway analysis demonstrated that circRNA-associated genes are involved in several key pathways, including ABC transporter activity, lysine degradation, regulation of the actin cytoskeleton, autophagy, focal adhesion, Rap1 signaling, and multiple cancer-related pathways (Figure 4E). Additionally, GO BP analysis of the differentially expressed mRNAs showed enrichment in developmental and differentiation processes such as keratinization, prostate gland (including prostatic acini) morphogenesis, and reproductive organ development (Figure 4D).

### Regulatory networks involving lncRNAs, circRNAs, miRNAs, and mRNAs

**FIGURE 5:**
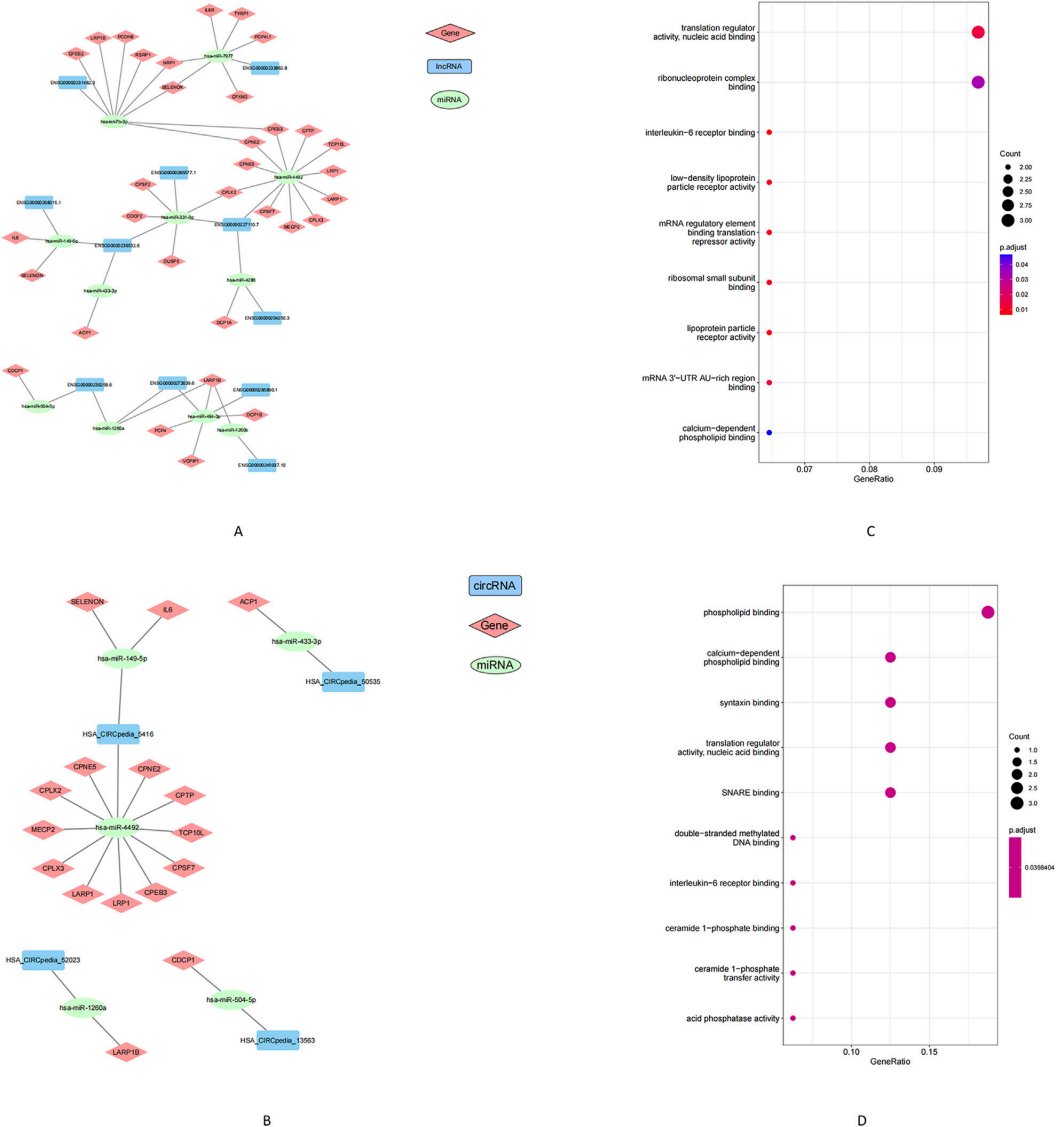
Analysis of the construction and GO enrichment of the ceRNA network. **(A)** lncRNA-miRNA-mRNA networks, **(B)** circRNA-miRNA-mRNA networks. **(C)** Gene enrichment in the lncRNA-miRNA-mRNA network. **(D)** Gene enrichment in the circRNA-miRNA-mRNA network. Note: Squares represent lncRNA or circRNA, circles represent miRNA, diamonds represent mRNA. Green indicates upregulation, blue or red indicates downregulation. Black lines indicate the correlation between lncRNA/circRNA-miRNA-mRNA.

ncRNAs can regulate gene expression by acting as competing ceRNAs that sequester miRNAs. Using this ceRNA framework, we constructed miRNA-centered interaction networks to examine how lncRNAs and circRNAs might modulate mRNA expression in POP. Differentially expressed (DE) lncRNAs, circRNAs, miRNAs, and mRNAs were integrated into a co-expression network based on predicted miRNA binding interactions (MiRanda and RNAhybrid) and significant inverse correlations between miRNA and target RNA levels. The final lncRNA–miRNA–mRNA network comprised 11 DE miRNAs, 11 DE lncRNAs, and 40 DE mRNAs (Figure 5A), whereas the circRNA–miRNA–mRNA network included 5 DE miRNAs, 4 DE circRNAs, and 16 DE mRNAs (Figure 5B). GO enrichment analysis of the mRNAs within these networks uncovered distinct functional categories. The lncRNA-associated network genes were enriched for MF such as interleukin-6 receptor binding and low-density lipoprotein particle receptor activity (Figure 5C). In the circRNA-associated network, the mRNAs were enriched in calcium-dependent phospholipid binding and synaptosomal fusion protein binding (Figure 5D). These enriched functions implicate pathways related to inflammatory signaling (IL-6 receptor pathways), metabolic regulation, and cellular membrane dynamics. Together, the ceRNA network findings highlight potential molecular mechanisms by which ncRNA–miRNA interactions may contribute to POP pathogenesis.

### Morphological identification of uterosacral ligament fibroblasts

**FIGURE 6:**
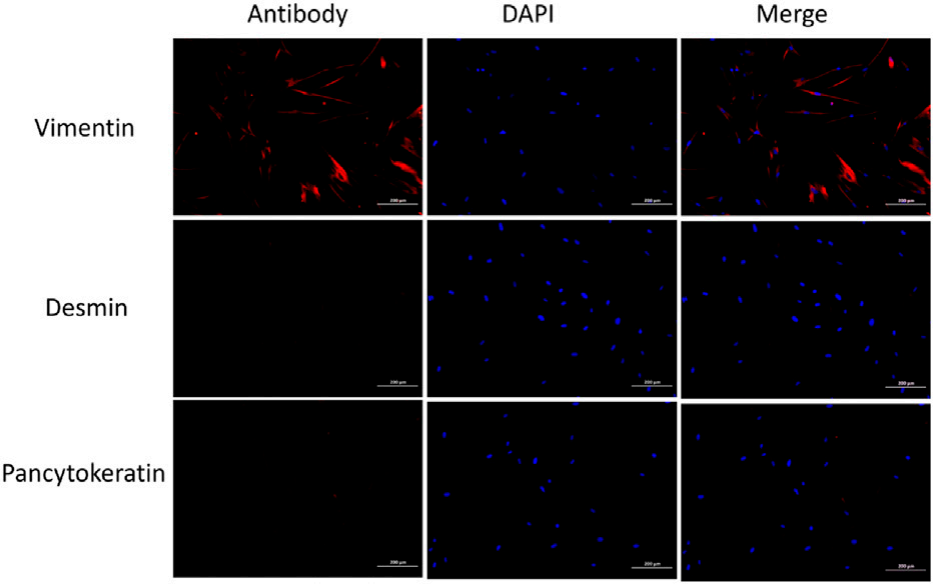
IF of fibroblasts shows positive for Vimentin, negative for desmin, and Pancytokeratin negative.

IF staining of cultured uterosacral ligament fibroblasts (passages 2 and 3) demonstrated robust expression of vimentin—a canonical fibroblast marker—while desmin and pancytokeratin signals were negligible, thus validating the fibroblastic identity of the isolated cells (Figure 6).

### Validation of candidate lncRNA

**FIGURE 7:**
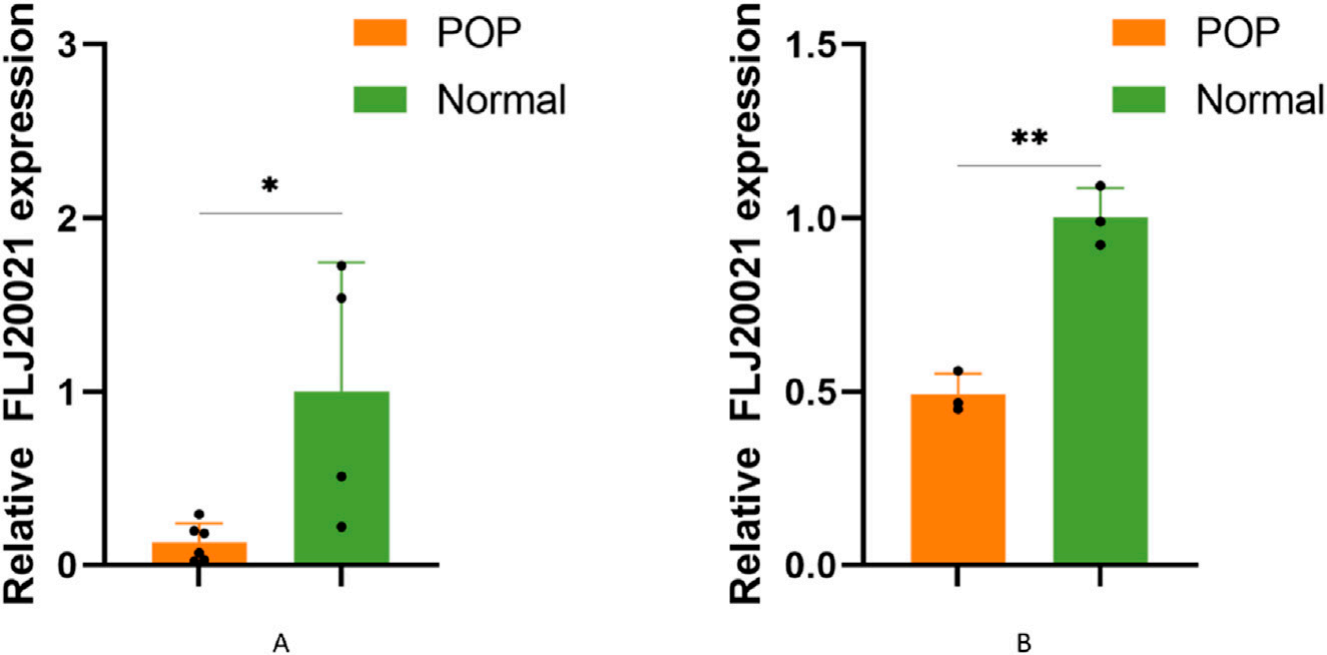
The relative expression of FLJ20021 were measured by qPCR in **(A)** uterosacral ligament tissues and **(B)** uterosacral ligament fibroblasts isolated from POP patients and age-matched normal controls. Data are presented as mean ± SD of three independent experiments. *P < 0.05, **P < 0.01 (Student’s t-test).

To explore the potential role of lncRNAs in the pathogenesis of POP, we screened for differentially expressed lncRNAs and identified lncRNA-FLJ20021 as a candidate of interest. qPCR was subsequently performed on uterosacral ligament tissues and cultured fibroblasts derived from both POP patients and normal controls. FLJ20021 expression was significantly reduced in the POP group compared with controls (Figures 7A, B), suggesting that downregulation of FLJ20021 may contribute to POP pathogenesis.

**FIGURE 8:**
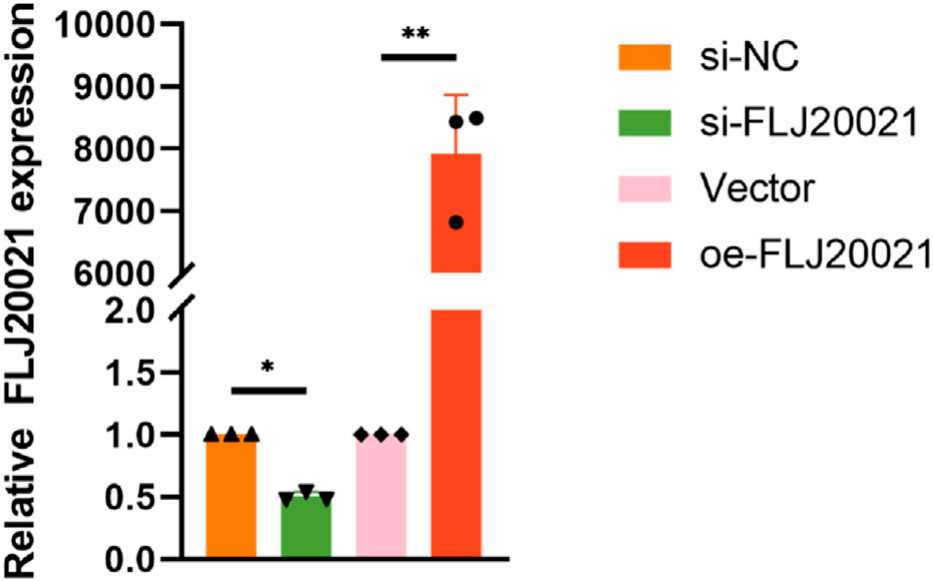
Efficiency of FLJ20021 knockdown and overexpression in fibroblasts.

**FIGURE 9:**
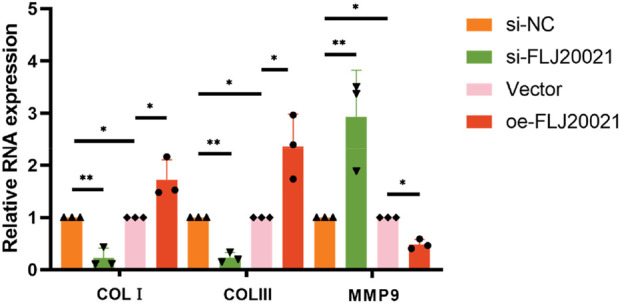
The effect of knocking down and overexpressing FLJ20021 on ECM by qPCR.

To elucidate the functional role of FLJ20021 in POP, we employed siRNAs to knockdown FLJ20021 in fibroblasts derived from POP and used plasmid-mediated transfection to achieve FLJ20021 overexpression. qPCR confirmed efficient modulation of FLJ20021 expression in fibroblasts (Figure 8). Subsequent analysis of key ECM components including COL III, COL I, and MMP9 revealed that FLJ20021 overexpression significantly increased COL III and COL I levels while reducing MMP9 expression. In contrast, FLJ20021 knockdown resulted in elevated MMP9 expression and concomitant decreases in COL III and COL I (Figure 9). These data suggest that FLJ20021 positively regulates ECM integrity, and its downregulation promotes ECM degradation in POP fibroblasts.

## Discussion

POP is a prevalent condition affecting nearly 50% of women over the age of 50 worldwide (Hw et al., 2022). Although rarely fatal, POP significantly impairs quality of life (Carroll et al., 2022). As a complex polygenic disorder, its pathogenesis is influenced by both epigenetic and environmental factors. Aberrant ECM and collagen metabolism (van Velthoven et al., 2024), imbalances between matrix metalloproteinases and their inhibitors (Kufaishi et al., 2016), and hormonal dysregulation (Rahn et al., 2024) all play crucial roles in POP. Moreover, specific cytokines and signaling pathways contribute to its development. An in-depth understanding of these molecular mechanisms may enhance diagnostic and prognostic capabilities and pave the way for novel preventive and therapeutic strategies.

Recent studies have increasingly focused on elucidating the pathogenic mechanisms underlying POP by analyzing disease-related gene sets. In our study, We performed comprehensive transcriptome profiling and identified widespread dysregulation of ncRNAs in POP tissues. We observed numerous differentially expressed lncRNAs, circRNAs, and miRNAs, highlighting a profound imbalance of the noncoding transcriptome in prolapsed versus normal pelvic tissue (Yu et al., 2022). Emerging evidence underscores the significance of ncRNAs in modulating fibroblast function and influencing POP outcomes (Lin et al., 2025; Wu et al., 2024). Specifically, we identified 29 differentially expressed lncRNAs (8 upregulated and 21 downregulated) in POP. KEGG enrichment analysis revealed significant involvement of the complement and coagulation cascades, TNF signaling, AGE–RAGE signaling, and ECM–receptor interactions—all pathways intimately linked to inflammatory responses, oxidative stress, and tissue remodeling. For example, extracellular matrix–receptor interactions are critical for maintaining the balance between ECM synthesis and degradation. Previous studies by Zhao et al. (2023) and Connell et al. (Ka et al., 2009) further support the role of altered lncRNA expression and downstream signaling pathways in POP pathogenesis. Despite these advances, our understanding of lncRNA regulation in POP remains limited.

Given the relative paucity of studies on certain lncRNAs, our investigation focused on the key lncRNA FLJ20021, located on chromosome 4q (NC_000004.12), which has been previously characterized as an oncogenic lncRNA in laryngeal cancer (Yin et al., 2024). We observed that FLJ20021 expression was significantly lower in POP tissues. *In vitro* experiments further confirmed that knockdown of FLJ20021 promoted ECM degradation, as evidenced by increased MMP9 and decreased collagen expression (Budatha et al., 2011). Its downregulation may contribute to the collagen degradation observed in POP, positioning FLJ20021 as a potential target for therapeutic intervention.

Previous investigations have also highlighted the contribution of miRNAs to POP, particularly those that post-transcriptionally regulate key ECM-related genes. For instance, miR-92 (19–25 nucleotides in length) regulates gene expression by binding to the 3′untranslated region (3′UTR) of target mRNAs, thereby inhibiting translation or promoting mRNA degradation (Yu et al., 2013). In our study, miR-92 expression was significantly elevated in the uterosacral ligaments of POP patients compared with controls. Furthermore, He et al. (2016) demonstrated through immunohistochemical analysis that miR-92 expression is inversely correlated with estrogen receptor β1 (ERβ1), indicating that miR-92 may contribute to the progression of POP. Current evidence indicates that estrogen receptor expression influences POP risk, with ERβ playing a role in regulating estrogen activity in fibroblasts (Suryazynski et al., 2003) and enhancing extracellular matrix synthesis (Zbucka-Kretowska et al., 2011). Additionally, the miR-29 family has been implicated in the suppression of elastin expression (Wang et al., 2023; Jin et al., 2016), and miR-19-3p appears to negatively regulate the Akt/mTOR/p70S6K pathway, leading to decreased COL I secretion (Yin et al., 2021). These findings further elucidate the molecular mechanisms contributing to POP development.

CircRNAs, which are generated by back-splicing, have recently gained attention for their roles in various cellular processes. However, the functions of both lncRNAs and circRNAs in POP remain relatively unexplored. Emerging evidence in other diseases suggests that lncRNAs and circRNAs serve as critical regulatory molecules, often acting as molecular sponges for miRNAs in accordance with the competing endogenous RNA (ceRNA) hypothesis (Yu et al., 2022). By integrating the expression profiles of lncRNAs, circRNAs, miRNAs, and mRNAs, we constructed a ceRNA network to elucidate potential interactions among these molecules. Our analysis revealed that dysregulated transcripts in POP were enriched in pathways related to inflammation, ECM remodeling, focal adhesion, and signal transduction—all fundamental processes for maintaining pelvic support. These results are consistent with those of (Yu et al. 2022), who identified disrupted focal adhesion signaling in POP via whole-transcriptome sequencing. For example, their ceRNA network included a module in which two circRNAs (hsa_circ_0002190 and hsa_circ_0046843) and an lncRNA (CARMN) sponged miR-23a-3p to regulate Rho-associated coiled-coil containing kinase isoform 2-a key regulator of actin cytoskeleton organization and smooth muscle contraction (Weber and Herskowitz, 2021). The convergence of similar pathway nodes in our network suggests that ncRNA disturbances in POP target common biological themes, particularly the disruption of cell–ECM interactions and mechanical signaling within fibroblasts. This is particularly relevant given that mechanical stress—stemming from factors such as childbirth trauma and chronic pressure—is a well-established precipitant of POP (Sun et al., 2024). Prolonged stretching of pelvic support ligaments may lead to progressive tissue deterioration, and our ceRNA network provides a molecular framework linking mechanical forces to altered gene expression and weakened ECM integrity.

The precise molecular mechanisms by which FLJ20021 regulates ECM components remain to be fully elucidated. Based on our ceRNA network analysis and established paradigms of lncRNA function, several mechanisms can be proposed. One possibility is that FLJ20021 functions as a ceRNA by sequestering miRNAs that normally target mRNAs encoding collagen, matrix metalloproteinases (MMPs), or their regulators. For example, if a miRNA typically suppresses MMP9 translation, FLJ20021 binding to this miRNA could lead to elevated MMP9 levels and enhanced collagen degradation. Alternatively, FLJ20021 might sequester a miRNA that promotes collagen expression by inhibiting a collagen repressor, thereby indirectly reducing collagen levels. Additionally, FLJ20021 may interact with protein factors or directly with target mRNAs in the nucleus to modulate gene transcription or mRNA stability. A recent study in laryngeal cancer demonstrated that FLJ20021 predominantly localizes to the nucleus and binds CDK1 mRNA, enhancing its stability (Yin et al., 2024). Although the context differs from POP, this observation suggests that FLJ20021 is capable of forming lncRNA–mRNA complexes that could similarly influence the expression of key ECM regulators in fibroblasts. These hypotheses warrant further experimental investigation.

This study has several limitations. The relatively small cohort of POP patients and controls necessitates validation in larger, more diverse populations to confirm that the identified ncRNA signatures are consistently associated with POP. Furthermore, our focus on FLJ20021-based on its prominence in our data-meant that other potentially important ncRNAs were not explored in equal detail. In addition, the role of FLJ20021 has not yet been validated in animal models or clinical settings, and direct evidence that modulating FLJ20021 can prevent or reverse POP is lacking. Similarly, the predicted interactions among other ncRNAs in our network require experimental confirmation.

To translate our transcriptomic insights into improved POP management, future studies should expand sample size and include diverse populations to ensure generalizability and robustness of the ncRNA signature, employ animal models to elucidate causal roles of key molecules such as FLJ20021, and integrate multi-omics approaches (proteomics, epigenomics, and metabolomics) to comprehensively map regulatory networks. Prospective longitudinal studies are needed to monitor ncRNA expression dynamics in relation to disease progression, while evaluating ncRNAs as noninvasive biomarkers for early detection, prognosis, and treatment monitoring.

In conclusion, our comprehensive transcriptomic analysis reveals significant dysregulation of ncRNAs in POP and highlights the potential role of FLJ20021 in maintaining ECM integrity. The constructed ceRNA network offers a systems-level perspective on POP pathogenesis and provides novel insights into the molecular interplay among ncRNAs, thereby identifying potential targets for future therapeutic intervention.

## Data Availability

The data presented in the study are deposited in the Figshare repository, accession DIO number: https://doi.org/10.6084/m9.figshare.29161949.v1.

## Abbreviations

POP, Pelvic organ prolapse; ncRNA, non-coding RNA; GO, Gene Ontology; KEGG, Kyoto Encyclopedia of Genes and Genomes; ceRNA, Competitive Endogenous RNA; MF, Molecular Functions; BP, Biological Processes; CC, Cellular Components; qPCR, Real-Time Fluorescent Quantitative PCR; DE, Differentially Expressed; miRNA, MicroRNA; lncRNA, Long Non-coding RNA; circRNA, Circular RNA; mRNA, Messenger RNA; BMI, Body Mass Index; IL-6, Interleukin-6; 3′UTR, 3′Untranslated RegionI; HC, Immunohistochemistry; COL Ⅰ, CollagenⅠ; COL Ⅲ, Collagen Ⅲ; MMP9, Matrix Metalloproteinase-9; ECM, Extracellular Matrix; padj, Adjusted P-value; MAPK, Mitogen-Activated Protein Kinase; Erk1/2, Extracellular Signal-Regulated Kinase 1/2; Rap1, Ras-related Protein 1; FOXO, Forkhead Box O; IF, Immunofluorescence; MMPs, Metalloproteinases.
